# Supplementary material for: Feasibility, acceptability and implementation of a whole-family mental health intervention for displaced adolescent girls in Colombia: A mixed-methods pilot randomized controlled trial
Source: Glob Ment Health (Camb). 2026 Feb 24;13:e47. doi: 10.1017/gmh.2026.10161 (PMC12973242; doi:10.1017/gmh.2026.10161)
Supplement: Seff et al. supplementary material 1 — Seff et al. supplementary material [file S2054425126101617sup001.docx]

CONSORT 2010 checklist of information to include when reporting a pilot or feasibility trial*


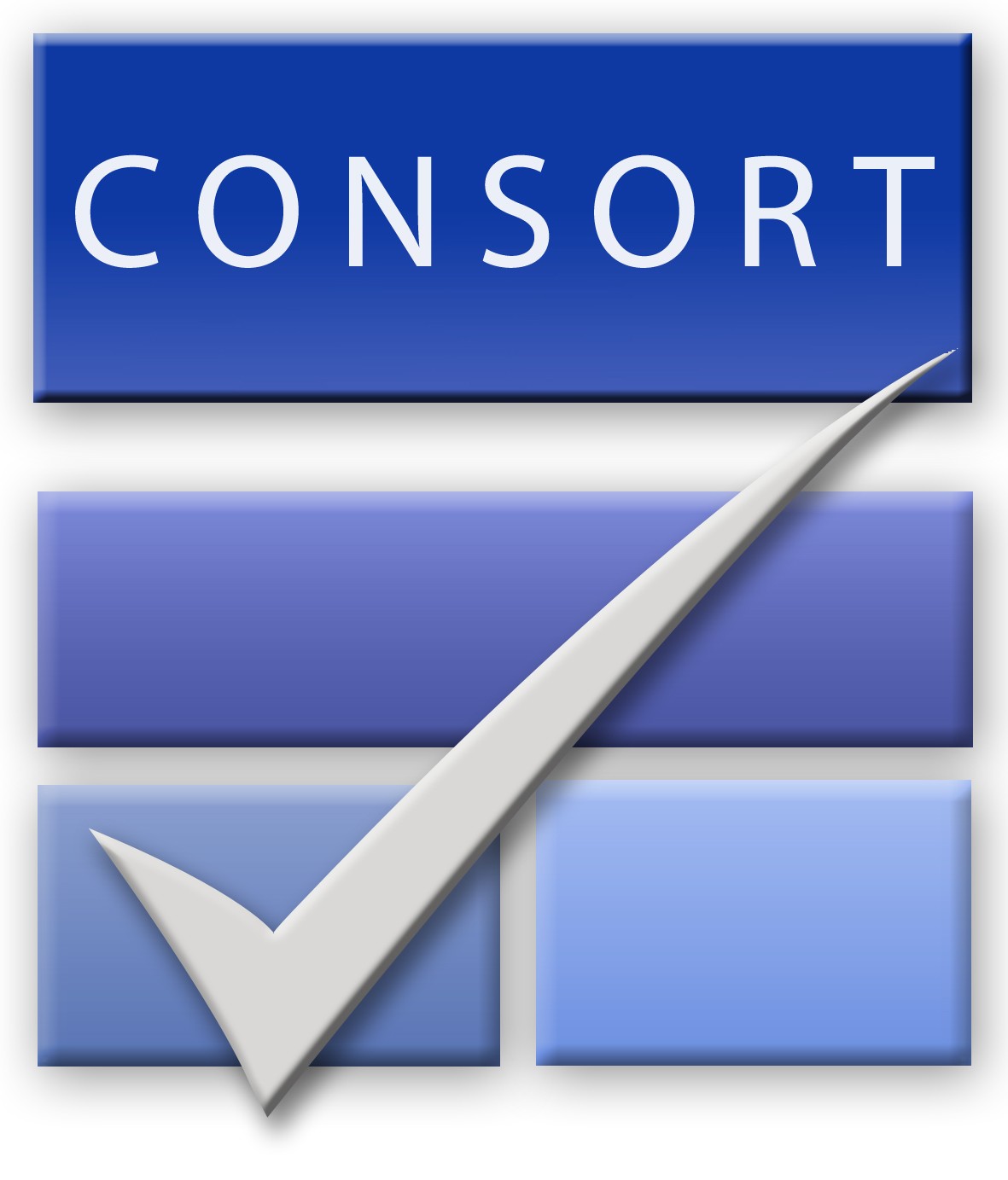


| Section/Topic | Item No | Checklist item | Reported on page No |
| --- | --- | --- | --- |
| Title and abstract | | | |
|  | 1a | Identification as a pilot or feasibility randomised trial in the title | Page 1, Title: "A Mixed-Methods Pilot Randomized Controlled Trial" |
|  | 1b | Structured summary of pilot trial design, methods, results, and conclusions (for specific guidance see CONSORT abstract extension for pilot trials) | Page 1, Abstract section (Background, Methods, Results, Conclusion) |
| Introduction | | | |
| Background and objectives | 2a | Scientific background and explanation of rationale for future definitive trial, and reasons for randomised pilot trial | Pages 2-4, Background section; Page 4: "This manuscript presents endline findings from a type-1 hybrid effectiveness-implementation mixed-methods pilot evaluation. This design was selected because SSAGE had demonstrated feasibility and acceptability …" |
|  | 2b | Specific objectives or research questions for pilot trial | Page 4, last paragraph of Background section: "We assess the potential impact on key mental health indicators... alongside key implementation outcomes, including acceptability, feasibility, and barriers and facilitators to program engagement" |
| Methods | | | |
| Trial design | 3a | Description of pilot trial design (such as parallel, factorial) including allocation ratio | Page 4: "type-1 hybrid effectiveness-implementation... pilot evaluation"; Methods section, page 5: "random number generator was used to randomize 93 girls each to the treatment or control arm in a 1:1 ratio" |
|  | 3b | Important changes to methods after pilot trial commencement (such as eligibility criteria), with reasons | Methods section, page 5: "Original eligibility criteria dictated that adolescent girls also live with an adult male caregiver... This requirement was removed after in-depth discussions" |
| Participants | 4a | Eligibility criteria for participants | Methods section, page 5: "Eligibility criteria included adolescent girls who had migrated from Venezuela... 13-19 years old, being available, along with their family members, to participate in the SSAGE intervention for twelve weeks" |
|  | 4b | Settings and locations where the data were collected | Page 7: "Venezuelan migrant and Colombian returnee families in Colombia"; Page 1, Abstract: mentions urban migrant settings |
|  | 4c | How participants were identified and consented | Methods section, page 4: "Participants for this study were recruited by Mercy Corps staff from a pool of families within Mercy Corps database"; detailed consent process described on page 5 |
| Interventions | 5 | The interventions for each group with sufficient details to allow replication, including how and when they were actually administered | Methods section, pages 3 and 4: Detailed description of SSAGE intervention including "12-week, gender-transformative intervention", session details, and parallel sessions; Page 4: Intervention processes including mentor training and fidelity monitoring |
| Outcomes | 6a | Completely defined prespecified assessments or measurements to address each pilot trial objective specified in 2b, including how and when they were assessed | Methods section, pages 6-8: Detailed descriptions of effectiveness outcomes (DSM-V cross-cutting, RCADS-25, RSES) and implementation outcomes (mhIST, qualitative interviews) |
|  | 6b | Any changes to pilot trial assessments or measurements after the pilot trial commenced, with reasons | Methods section, page 6: "A modified version of the Family Attachment Changeability Index 8 (FACI-8)... demonstrated poor reliability... and was not included in the present evaluation" |
|  | 6c | If applicable, prespecified criteria used to judge whether, or how, to proceed with future definitive trial | Not applicable |
| Sample size | 7a | Rationale for numbers in the pilot trial | Methods section, page 5: "Additional details on sample size calculations have been published elsewhere, though it is important to note that this pilot trial is not powered to detect effectiveness" |
|  | 7b | When applicable, explanation of any interim analyses and stopping guidelines | Not applicable |
| Randomisation: |  |  |  |
| Sequence  generation | 8a | Method used to generate the random allocation sequence | Methods section, page 5: "A random number generator was used to randomize 93 girls each to the treatment or control arm" |
|  | 8b | Type of randomisation(s); details of any restriction (such as blocking and block size) | Methods section, page 5: "in a 1:1 ratio. Randomization was not blinded" |
| Allocation  concealment  mechanism | 9 | Mechanism used to implement the random allocation sequence (such as sequentially numbered containers), describing any steps taken to conceal the sequence until interventions were assigned | Not applicable |
| Implementation | 10 | Who generated the random allocation sequence, who enrolled participants, and who assigned participants to interventions | Methods section, page 5: “Consent and enrolment processes were carried out by the data collection team…” “Randomization was carried out by the research team and was not blinded” |
| Blinding | 11a | If done, who was blinded after assignment to interventions (for example, participants, care providers, those assessing outcomes) and how | Methods section, page 5: "Randomization was not blinded" |
|  | 11b | If relevant, description of the similarity of interventions | Not applicable |
| Statistical methods | 12 | Methods used to address each pilot trial objective whether qualitative or quantitative | Methods section, pages 7 and 8: Quantitative methods (linear and logistic regression); Qualitative methods (thematic analysis using Dedoose) |
| Results | | | |
| Participant flow (a diagram is strongly recommended) | 13a | For each group, the numbers of participants who were approached and/or assessed for eligibility, randomly assigned, received intended treatment, and were assessed for each objective | Results section, page 8: "186 adolescent girls were enrolled... 93 girls each were then randomly assigned"; "158 (85%) adolescent girls completed the endline survey"; Figure 1 (CONSORT flow diagram) referenced |
|  | 13b | For each group, losses and exclusions after randomisation, together with reasons | Results section, page 8: "158 (85%) adolescent girls completed the endline survey in December 2024, with equal loss-to-follow-up in both the treatment and control groups" |
| Recruitment | 14a | Dates defining the periods of recruitment and follow-up | Results section, page 8: "In August 2024, 186 adolescent girls were enrolled"; "completed the endline survey in December 2024" |
|  | 14b | Why the pilot trial ended or was stopped | Not applicable |
| Baseline data | 15 | A table showing baseline demographic and clinical characteristics for each group | Page 24: Table 1. Baseline characteristics |
| Numbers analysed | 16 | For each objective, number of participants (denominator) included in each analysis. If relevant, these numbers  should be by randomised group | Results section, page 9: "88 of the 93 families assigned to the treatment arm" for attendance data |
| Outcomes and estimation | 17 | For each objective, results including expressions of uncertainty (such as 95% confidence interval) for any  estimates. If relevant, these results should be by randomised group | Page 25: Table 2 shows beta coefficients with 95% confidence intervals; Pages 9-12: Implementation findings with quantitative and qualitative results |
| Ancillary analyses | 18 | Results of any other analyses performed that could be used to inform the future definitive trial | Result section, page 8: "Additionally, no outcomes of interest were found to be associated with loss-to-follow up"; Pages 9-12: Extensive implementation evaluation findings |
| Harms | 19 | All important harms or unintended effects in each group (for specific guidance see CONSORT for harms) | No harms occurred |
|  | 19a | If relevant, other important unintended consequences | Not applicable |
| Discussion | | | |
| Limitations | 20 | Pilot trial limitations, addressing sources of potential bias and remaining uncertainty about feasibility | Discussion section, page 14: Detailed discussion of limitations including low attendance, self-reported outcomes, cultural validity of measures, and generalizability |
| Generalisability | 21 | Generalisability (applicability) of pilot trial methods and findings to future definitive trial and other studies | Discussion section, page 14: "findings from the study may not be generalizable for camp-based or rural settings" |
| Interpretation | 22 | Interpretation consistent with pilot trial objectives and findings, balancing potential benefits and harms, and  considering other relevant evidence | Discussion section, pages 12 and 13: Comprehensive discussion of findings |
|  | 22a | Implications for progression from pilot to future definitive trial, including any proposed amendments | Pages 20-21: Detailed recommendations including increasing monetary incentives, adding income-generating activities, and flexible scheduling |
| Other information | | |  |
| Registration | 23 | Registration number for pilot trial and name of trial registry | Methods section, page 5: "The trial was registered with ClinicalTrials as # NCT06078124" |
| Protocol | 24 | Where the pilot trial protocol can be accessed, if available | Background section, page 3: " Additional details on the intervention’s protocol and theory of change have been published elsewhere (Seff et al. 2024)" |
| Funding | 25 | Sources of funding and other support (such as supply of drugs), role of funders | Page 14: "This research was funded by the National Institute of Health (R34MH134078)" |
|  | 26 | Ethical approval or approval by research review committee, confirmed with reference number | Page 15: "All study procedures received ethical approval from the Institutional Review Board at the University of Los Andes and Washington University in St. Louis" (#202407174) |

Citation: Eldridge SM, Chan CL, Campbell MJ, Bond CM, Hopewell S, Thabane L, et al. CONSORT 2010 statement: extension to randomised pilot and feasibility trials. BMJ. 2016;355. This is an Open Access article distributed in accordance with the terms of the Creative Commons Attribution (CC BY 3.0) license (<http://creativecommons.org/licenses/by/3.0/>), which permits others to distribute, remix, adapt and build upon this work, for commercial use, provided the original work is properly cited.

*We strongly recommend reading this statement in conjunction with the CONSORT 2010, extension to randomised pilot and feasibility trials, Explanation and Elaboration for important clarifications on all the items. If relevant, we also recommend reading CONSORT extensions for cluster randomised trials, non-inferiority and equivalence trials, non-pharmacological treatments, herbal interventions, and pragmatic trials. Additional extensions are forthcoming: for those and for up-to-date references relevant to this checklist, see [www.consort-statement.org](http://www.consort-statement.org).
