# Supplementary material for: Feasibility, acceptability and implementation of a whole-family mental health intervention for displaced adolescent girls in Colombia: A mixed-methods pilot randomized controlled trial
Source: Glob Ment Health (Camb). 2026 Feb 24;13:e47. doi: 10.1017/gmh.2026.10161 (PMC12973242; doi:10.1017/gmh.2026.10161)
Supplement: Seff et al. supplementary material 2 — Seff et al. supplementary material [file S2054425126101617sup002.docx]

***Thematic Axes of the Girls' Curriculum***

| **SESSION 1 THIS IS ME!** |
| --- |
| - Activity 1.1 Dynamic activity (spider web) |
| - Activity 1.2 This is me! |
| - Activity 1.3 Group agreements |
| - Activity 1.4 Closing the session |
| **SESSION 2 WHAT DOES IT MEAN TO BE A GIRL? (PART 1)** |
| - Welcome and summary |
| - Activity 2.1 How we learn to be girls (and boys) |
| - Activity 2.2 Girl box, boy box |
| - Activity 2.3 Sex and gender |
| - Activity 2.4 Closing the session |
| **SESSION 3 WHAT DOES IT MEAN TO BE A GIRL? (PART 2)** |
| - Welcome and summary |
| - Activity 3.1 Gender values |
| - Activity 3.2 The work we do and the value given to it |
| - Activity 3.3 Closing the session |
| **SESSION 4: POWER AND EMPOWERMENT** |
| - Important note for mentors regarding girls' comfort and safety |
| - Welcome and summary |
| - Activity 4.1 Balance of power |
| - Activity 4.2 The circle of discrimination |
| - Activity 4.3 This is us! |
| - Activity 4.4 Closing the session |
| **SESSION 5: WHAT IS VIOLENCE?** |
| - Welcome and summary |
| - Activity 5.1 Types of violence |
| - Activity 5.2 Consequences of gender violence |
| - Activity 5.3 Who are the perpetrators of gender violence? |
| - Activity 5.4 Closing the session |
| **SESSION 6: STAYING SAFE FROM VIOLENCE** |
| - Welcome and summary |
| - Activity 6.1 Violence Identification Test |
| - Activity 6.2 Staying safe from violence |
| - Activity 6.3 My safety net |
| - Activity 6.4 Closing the session |
| **SESSION 7: MEETING 1: MY BODY, MY RIGHTS** |
| - Welcome and summary |
| - Activity 7.1 Physical and emotional changes in boys and girls |
| - Activity 7.2 Our rights |
| - Activity 7.3 Reproductive myths |
| - Activity 7.4 Closing the session |
| **SESSION 7: MEETING 2: MY EMOTIONAL WELL-BEING AND GOOD FAMILY TREATMENT** |
| - Welcome and summary |
| - Activity 7.1: Emotions, their sensations and ways of |
| - Activity 7.2: Care routes – 15 minutes. |
| - Activity 7.3: We manage emotions and solve problems |
| - Activity 7.4: Closing the session |
| **SESSION 8: USE AND ABUSE OF ALCOHOL AND OTHER SUBSTANCES** |
| - Welcome and summary |
| - Activity 8.1 What do we know about drugs? |
| - Activity 8.2 Drugs in our lives and communities |
| - Activity 8.3 Closing of the session |
| **SESSION 9: HEALTHY RELATIONSHIPS** |
| - Welcome and summary |
| - Activity 9.1 My relationships |
| - Activity 9.2 Healthy and unhealthy relationships |
| - Activity 9.3 Family relationships |
| - Activity 9.4 Closing the session |
| **SESSION 10: INTERPERSONAL COMMUNICATION** |
| - Welcome and summary |
| - Activity 10.1 Listening Skills |
| - Activity 10.2 Being assertive |
| - Activity 10.3 Resolving disagreements |
| - Activity 10.4 Closing the session |
| **SESSION 11: DECISION MAKING** |
| - Welcome and summary |
| - Activity 11.1 Personal decision-making |
| - Activity 11.2 Resisting influences/moving forward |
| - Activity 11.3 Closing the session |
| **SESSION 12: LOOKING FORWARD** |
| - Welcome and summary |
| - Activity 12.1 This is me (part 2)! |
| - Activity 12.2 I promise… |
| - Activity 12.3 The spider web |
| - Activity 12.4 Closing the session |

**Thematic Axes Children's Curriculum**

| **SESSION 1 WHAT DOES IT MEAN TO BE A MAN?** |
| --- |
| Activity 1.1: Icebreaker ( spider web ) |
| Activity 1.2: Agreements group |
| Activity 1.3: What does it mean to be a man in society? |
| Activity 1.4: Closing the session |
| **SESSION 2: GENDER SOCIALIZATION** |
| Welcome and summary |
| Activity 2.1: How we learn to be boys (and girls) |
| Activity 2.2: Boy box, girl box |
| Activity 2.3: Sex and gender |
| Activity 2.4: Closing the session |
| **SESSION 3 GENDER ROLES** |
| Welcome and summary |
| Activity 3.1: Gender values |
| Activity 3.2: The work we do and the value placed on it |
| Activity 3.3: Closing the session |
| **SESSION 4: POWER AND DISCRIMINATION** |
| Welcome and summary |
| Activity 4.1: Balance of power |
| Activity 4.2: Building a new world |
| Activity 4.3: The circle of discrimination |
| Activity 4.4: Closing the session |
| **SESSION #5: TYPES OF VIOLENCE** |
| Welcome and summary |
| Activity 5.1 Children 's games |
| Activity 5.2 Violence in our lives |
| Activity 5.3 Closing the session |
| **SESSION 6: GENDER VIOLENCE** |
| Welcome and summary |
| Activity 6.1 Who uses violence and why |
| Activity 6.2 Consequences of gender violence |
| Activity 6.3 The cycle of domestic violence |
| Activity 6.4 Closing the session |
| **SESSION 7: PREVENTION OF VG** |
| Welcome and summary |
| Activity 7.1 Violence in daily life |
| Activity 7.2 Taking a stand against violence |
| Activity 7.3 The map of power and violence |
| Activity 7.4 Closing the session |
| **SESSION 7 (MEETING 2): MY EMOTIONAL WELL-BEING AND GOOD FAMILY TREATMENT** |
| Welcome and summary |
| Activity 7.1: Emotions |
| Activity 7.2: Care routes |
| Activity 7.3: We manage emotions and solve problems |
| Activity 7.4: Closing the session |
| **SESSION 8: USE AND ABUSE OF ALCOHOL AND OTHER SUBSTANCES** |
| Welcome and summary |
| Activity 8.1 What do we know about drugs? |
| Activity 8.2 Drugs in our lives and communities |
| Activity 8.3 Closing the session |
| **SESSION 9: HEALTHY RELATIONSHIPS** |
| Welcome and summary |
| Activity 9.1 Love and romance |
| Activity 9.2 Consent |
| Activity 9.3 Healthy and unhealthy relationships |
| Activity 9.4 Closing the session |
| **SESSION 10: INTERPERSONAL COMMUNICATION** |
| Welcome and summary |
| Activity 10.1 Listening Skills​ |
| Activity 10.2 Being assertive |
| Activity 10.3 Resolving disagreements |
| Activity 10.4 Closing the session |
| **SESSION 11: DECISION MAKING** |
| Welcome and summary |
| Activity 11.1 Personal decision-making |
| Activity 11.2 Resisting influences/moving forward |
| Activity 11.3 Closing the session |
| **SESSION 12: LOOKING FORWARD** |
| Welcome and summary |
| Activity 12.1 What does it REALLY mean to be a man? |
| Activity 12.2 I promise … |
| Activity 12.3 The spider web |
| Activity 12.4 Closing the session |

**Thematic axes Curriculum Mothers and/or caregivers**

| **SESSION 1: THIS IS ME!** |
| --- |
| Activity 1.1: Dynamic activity (spider web) |
| Activity 1.2: This is me! |
| Activity 1.3: Group agreements |
| Activity 1.4: Closing the session |
| **SESSION 2: WHAT DOES IT MEAN TO BE A WOMAN? (PART 1)** |
| Welcome and summary |
| Activity 2.1: Gender values |
| Activity 2.2: Women's Box, Men's Box |
| Activity 2.3: Sex and gender |
| Activity 2.4: Closing the session |
| **SESSION 3: WHAT DOES IT MEAN TO BE A WOMAN? (PART 2)** |
| Welcome and summary |
| Activity 3.1: How we learn to be women (and men) |
| Activity 3.2: The work we do and the value placed on it |
| Activity 3.3: Closing the session |
| **SESSION 4: POWER AND EMPOWERMENT** |
| Important note for mentors regarding women's comfort and safety |
| Welcome and summary |
| Activity 4.1 Balance of power |
| Activity 4.2: Exploring the meaning of power |
| Activity 4.3: Who has power and how they use it |
| Activity 4.4: The circle of discrimination |
| Activity 4.5: Closing the session |
| **SESSION 5: WHAT IS VIOLENCE?** |
| Welcome and summary |
| Activity 5.1: Types of violence |
| Activity 5.2: The cycle of domestic violence |
| Activity 5.3: Consequences of gender violence |
| Activity 5.4: Closing the session |
| **SESSION 6: STAYING SAFE FROM VIOLENCE** |
| Welcome and summary |
| Activity 6.1: Violence in daily life |
| Activity 6.2: Staying safe from violence |
| Activity 6.3: The map of power and violence |
| Activity 6.4: Closing the session |
| **SESSION 7 MEETING 1: MY EMOTIONAL WELL-BEING AND GOOD FAMILY TREATMENT** |
| Activity 7.1: Emotions, their sensations and forms of expression |
| Activity 7.2: Care routes |
| Activity 7.3: We manage emotions in good family treatment. |
| Activity 7.4 Closing the session |
| **SESSION 7 MEETING 2: HEALTHY COUPLE RELATIONSHIPS** |
| Welcome and summary |
| Activity 7.1: My Relationships |
| Activity 7.2: Healthy Relationships |
| Activity 7.3: Healthy and unhealthy relationships |
| Activity 7.4 Closing the session |
| **SESSION 8: ADOLESCENCE** |
| Activity 8.1 Physical and emotional changes in boys and girls |
| Activity 8.2 Reproductive myths |
| Activity 8.3 Rights of adolescent girls |
| Activity 8.4 Closing the session |
| **SESSION 9 TEENAGE GIRLS AND VG** |
| Welcome and summary |
| Activity 9.1 Understanding violence against adolescent girls |
| Activity 9.2 Consequences of violence for adolescents |
| Activity 9.3 Protecting adolescent girls from violence |
| Activity 9.4 Closing the session |
| **SESSION 10 LISTENING AND COMMUNICATION** |
| Welcome and summary |
| Activity 10.1 Listening Skills |
| Activity 10.2 Communication between mother and children |
| Activity 10.3 Empathy between mothers and daughters (and sons) |
| Activity 10.4 Closing of the session |
| **SESSION 11 HEALTHY FAMILY ENVIRONMENT** |
| Welcome and summary |
| Activity 11.1 Family Rules and Expectations |
| Activity 11.2 Decisions at home |
| Activity 11.3 Resolving disagreements |
| Activity 11.4 Closing the session |
| **SESSION 12 POSITIVE PARENTING** |
| Welcome and summary |
| Activity 12.1 My parents' legacy |
| Activity 12.2 Positive parenting techniques |
| Activity 12.3 Closing the session |
| Activity 12.4 Closing the session |
| **SESSION 13 LOOKING FORWARD** |
| Welcome and summary |
| Activity 13.1 This is me (part 2)! |
| Activity 13.2 I promise… |
| Activity 13.3 The spider web |

**Curriculum Themes for Parents and/or Caregivers**

| **SESSION 1: WHAT DOES IT MEAN TO BE A MAN?** |
| --- |
| Activity 1.1 Icebreaker (spider web) |
| Activity 1.2 Group agreements |
| Activity 1.3 What does it mean to be a man in society? |
| Activity 1.4 Closing the session |
| **SESSION 2: GENDER SOCIALIZATION** |
| Welcome and summary |
| Activity 2.1 How we learn to be men (and women) |
| Activity 2.2 Men's box, Women's box |
| Activity 2.3 Sex and gender |
| Activity 2.4 Closing the session |
| **SESSION 3: GENDER ROLES** |
| Welcome and summary |
| Activity 3.1: Gender values |
| Activity 3.2: The work we do and the value given to it |
| Activity 3.3 Closing the session |
| **SESSION 4: POWER AND DISCRIMINATION** |
| Welcome and summary |
| Activity 4.1 Balance of power |
| Activity 4.2 Building a new world |
| Activity 4.3 The circle of discrimination |
| Activity 4.4 Closing the session |
| **SESSION 5: TYPES OF VIOLENCE** |
| Welcome and summary |
| Activity 5.1 Children's games |
| Activity 5.2 Violence in our lives |
| Activity 5.3 Closing the session |
| **SESSION 6: GENDER-BASED VIOLENCE (GBV)** |
| Welcome and summary |
| Activity 6.1 Who uses violence and why? |
| Activity 6.2 Consequences of gender violence |
| Activity 6.3 The cycle of domestic violence |
| Activity 6.4 Closing the session |
| **SESSION 7 MEETING 1: GBV PREVENTION** |
| Welcome and summary |
| Activity 7.1 Violence in daily life |
| Activity 7.2 Taking a stand against violence |
| Activity 7.3 The map of power and violence |
| Activity 7.4 Closing the session |
| **SESSION 7 MEETING 2: MY EMOTIONAL WELL-BEING AND GOOD FAMILY TREATMENT** |
| Welcome and summary |
| Activity 7.1: Emotions, their sensations and forms of expression |
| Activity 7.2: Care routes |
| Activity 7.3: Managing emotions in good family relationships |
| Activity 7.4: Closing the session |
| **SESSION 8: HEALTHY RELATIONSHIPS** |
| Welcome and summary |
| Activity 8.1 My relationships |
| Activity 8.2 Healthy and unhealthy relationships |
| Activity 8.3 Consent |
| Activity 8.4 Closing the session |
| **SESSION 9: ADOLESCENCE** |
| Activity 9.1 Physical and emotional changes in boys and girls |
| Activity 9.2 Parent-child relationships |
| Activity 9.3 Rights of adolescent girls |
| Activity 9.4 Closing the session |
| **SESSION 10: PROTECTING ADOLESCENT GIRLS AGAINST VG** |
| Welcome and summary |
| Activity 10.1 Understanding violence against adolescent girls |
| Activity 10.2 Consequences of violence for adolescents |
| Activity 10.3 Protecting adolescent girls from violence |
| Activity 10.4 Closing the session |
| **SESSION 11: INTERPERSONAL COMMUNICATION** |
| Welcome and summary |
| Activity 11.1 Listening Skills |
| Activity 11.2 Empathy between parents and their children |
| Activity 11.3 Resolving disagreements |
| Activity 11.4 Closing the session |
| **SESSION 12: HEALTHY FAMILY ENVIRONMENT** |
| Welcome and summary |
| Activity 12.1 Family Rules and Expectations |
| Activity 12.2 Decisions at home |
| Activity 12.3 Positive parenting techniques |
| Activity 12.4 Closing the session |
| **SESSION 13: LOOKING FORWARD** |
| Welcome and summary |
| Activity 13.1 What does it REALLY mean to be a man? |
| Activity 13.2 I promise… |
| Activity 13.3 The spider web |
| Activity 13.4 Closing the session |
